# Optimal Indicator of Death for Using Real-World Cancer Patients' Data From the Healthcare System

**DOI:** 10.3389/fphar.2022.906211

**Published:** 2022-06-16

**Authors:** Suk-Chan Jang, Sun-Hong Kwon, Serim Min, Ae-Ryeo Jo, Eui-Kyung Lee, Jin Hyun Nam

**Affiliations:** ^1^ School of Pharmacy, Sungkyunkwan University, Suwon, South Korea; ^2^ Divison of Big Data Science, Korea University Sejong Campus, Sejong, South Korea

**Keywords:** optimal indicator of death, cancer patients, operational definition of death, claims data, real-world data

## Abstract

**Background:** Information on patient’s death is a major outcome of health-related research, but it is not always available in claim-based databases. Herein, we suggested the operational definition of death as an optimal indicator of real death and aim to examine its validity and application in patients with cancer.

**Materials and methods:** Data of newly diagnosed patients with cancer between 2006 and 2015 from the Korean National Health Insurance Service—National Sample Cohort data were used. Death indicators were operationally defined as follows: 1) in-hospital death (the result of treatment or disease diagnosis code from claims data), or 2) case wherein there are no claims within 365 days of the last claim. We estimated true-positive rates (TPR) and false-positive rates (FPR) for real death and operational definition of death in patients with high-, middle-, and low-mortality cancers. Kaplan−Meier survival curves and log-rank tests were conducted to determine whether real death and operational definition of death rates were consistent.

**Results:** A total of 40,970 patients with cancer were recruited for this study. Among them, 12,604 patients were officially reported as dead. These patients were stratified into high- (lung, liver, and pancreatic), middle- (stomach, skin, and kidney), and low- (thyroid) mortality groups consisting of 6,626 (death: 4,287), 7,282 (1,858), and 6,316 (93) patients, respectively. The TPR was 97.08% and the FPR was 0.98% in the high mortality group. In the case of the middle and low mortality groups, the TPR (FPR) was 95.86% (1.77%) and 97.85% (0.58%), respectively. The overall TPR and FPR were 96.68 and 1.27%. There was no significant difference between the real and operational definition of death in the log-rank test for all types of cancers except for thyroid cancer.

**Conclusion:** Defining deaths operationally using in-hospital death data and periods after the last claim is a robust alternative to identifying mortality in patients with cancer. This optimal indicator of death will promote research using claim-based data lacking death information.

## Introduction

Over the decades, abundant data have been produced and used in various fields, including health-related data. The term ‘Real-World Data (RWD)’ has been introduced with the advent of the big data era, which includes data about patients’ health status, health care utilization, or cost collected from sources other than traditional clinical trials. RWD consists of electronic health records, claims and billing data, and registries among others (U.S. Food and Drug Administration, 2017; 2020; 2021). There has been an increasing demand to use RWD as a substitute for clinical trial data under the 21st Century Cures Act, which provides guidance on how RWD can influence decision-making, including label expansion for approved products and post-market commitments (United States Congress legislative information, 2016).

Survival is the most direct indicator of a patient’s health status and has a critical impact on health-related decision-making. For this reason, survival-associated outcomes are presented as the major outcomes in most clinical studies, including clinical trials and epidemiological studies; they are also key parameters in studies of health policy, health economics, and outcomes research (Podrid and Myerburg, 2005; Khera et al., 2021; Sanyal et al., 2021). However, death is the most difficult outcome to observe in studies with short-term follow-up. Many clinical trials are not conducted for sufficiently long durations to estimate survival rates, and immature survival data increase the uncertainty in cost-effectiveness studies (Tai et al., 2021). Since the confirmation of death is critical, especially in research targeting severe disease, death information needs to be underpinned by studies using long-term observational data.

RWD, especially insurance claims data, has been considered a valuable resource in health-related research. Although claims data have provided abundant information about patients, including demographics, disease diagnosis codes, and prescription drugs, the patients’ death information is not provided in many types of claims data. The National Database of Health Care Claims from Japan, or many United States claims databases were not linked to death information (Ministry of Health, Labour and Welfare, Japan, 2018; Reps et al., 2019; Yasunaga, 2019). Moreover, the patient’s death information is limited to the claims data from the Health Insurance Review & Assessment Service, which is most widely used in South Korea (Korean Health Insurance Review and Assessment Service, 2021). The lack of these may pose serious challenges such as censoring in using claims data for health-related research (Johansson and Westerling, 2000; Calvo-Alen et al., 2005).

To circumvent this limitation and investigate the overall survival rates in claims data, many studies have adopted their own definitions for the suspicious indicator for death (Yuk et al., 2016; Shim et al., 2020). However, in these cases, the overall survival rates are likely to be underestimated, as they reported. In this study, we suggested an alternative definition of death that can function as an optimal indicator of real death to investigate the overall survival rates using claims data in which patients’ death information is not provided. Since cancer is a disease that is closely related to mortality, we applied this definition to data from groups of patients with cancer, who were stratified based on cancer types as per the mortality rates, for validation.

## Materials and Methods

### Data Source

We used the Korean National Health Insurance Service-National Sample Cohort (NHIS-NSC) data, the representativeness of which has been verified (Lee et al., 2017). The NHIS database was established for patients’ health insurance reimbursements and contained all the information on patient demographic characteristics, disease diagnosis codes, prescription drugs, healthcare resource utilization, and medical expenditures. Codes for disease diagnosis were identified according to the International Statistical Classification of Diseases and Related Health Problems, 10th Revision (ICD-10, 2016). The NHIS-NSC included data from 2002 to 2015 of approximately 1 million randomly selected Koreans, representing 2% of the Korean population in 2006. The NHIS-NSC is linked to Statistics Korea; thus, it provides official death-related information, such as time and cause of death, making it ideal for this study.

### Study Population and Design

In this study, we recruited patients newly diagnosed with cancer having an ICD-10 code of “C” and a critical condition code for cancer (V193 or V194). Since the mortality rates are quite different for different cancer types, we carefully considered several types of cancers and grouped them according to the 5-year relative survival of each cancer as follows: the high-mortality group with 20% less survival (lung [C33 or C34], liver [C22], and pancreatic cancer [C25]), the middle-mortality group with 30–80% of survival (stomach [C16], skin [C43 or C44], and kidney cancer [C64 or C65]), and the low-mortality group with about 98% of survival (thyroid cancer [C73]) (Cancer Research UK, 2014; American Cancer Society, 2018; Bertuccio et al., 2019; Statistics Korea, 2021a). Additionally, all cancer patients not specified with cancer type were considered for presenting the overall trends. The one diagnosed earliest was used to determine their type in patients with multiple cancers. For this study, the cohort entry period was set from 1 January 2006 to 31 December 2015. The cohort entry date was defined as the first day of cancer diagnosis during the cohort entry period. Patients diagnosed with cancer within 365 days before the cohort entry date were excluded from the study, retaining only the newly diagnosed patients. The target patients were followed up until death or till the end of the study (31 December 2015), whichever occurred first.

### Operational Definition of Death

The combination of in-hospital death and the length of a period without medical utilization after the last claim was operationally defined as an indicator of death (ODD, Table 1). Since the claims data were based on treatment reimbursement, in-hospital death information was provided as a consequence of treatment, which can be identified as follows: 1) death indication as a result of treatment or 2) the ICD-10 codes I461, R96, R98, or R99. These ICD-10 codes have been used to indicate death in previous studies (Shin et al., 2015; Noh et al., 2016; Mentzer et al., 2018). The date of in-hospital death was defined as the date of claim on which the code was recorded. To further observe deaths not recorded as in-hospital deaths, we identified them as cases where there were no claims after the last claim. Several studies (Mealing et al., 2012; Lee et al., 2019) operationally defined death as the cases of no claims for 90 or 180 days. We considered 90/180/270/365 days as the length of a period without any claims. In this case, the date of death was defined as the date on the last claim that identified the patient as dead.

**TABLE 1:** The operational definition of death in claims data.

Type	Description	Date of death
In-hospital death		The date of claim when the code was recorded
(1) Treatment result	The case in which the result of treatment is coded as death	
(2) Disease codes	The case in which the claims data include at least one of the ICD-10 codes	
	– I461 (Sudden cardiac death)	
	– R96 (Other sudden death, cause unknown)	
	– R98 (Unattended death)	
	– R99 (Other ill-defined and unspecified cause of mortality)	
Length of the period without any claims	The case in which there are no claims within 365 days of the last claim. That is, there is none of the medical utilization over 365 days	The date of the last claim

### Validity of Operational Definition of Death

We estimated its true-positive rate (TPR) and false-positive rate (FPR) using real death data as the gold standard to validate the ODD. A true positive (TP) means that a dead patient is correctly identified as deceased, whereas a false positive (FP) implies that an alive patient is incorrectly identified as deceased. In contrast, true negative (TN) means that alive patients are correctly identified as alive and false negative (FN) means that dead patients are incorrectly identified as alive. TPR is calculated as TPR = TP/(TP + FN) and represents the proportion of patients who were designated as dead by ODD out of the officially dead patients. Additionally, FPR is determined as FPR = FP/(TN + FP) and represents the proportion of patients who were incorrectly classified as dead by ODD out of the patients alive. TPR and FPR refer to sensitivity and 1-specificity, respectively, and are adequate measures for testing the consistency between real and operational deaths. Based on the estimated TPR and FPR according to the length of periods (90/180/270/365 days), Another measure to validate the usefulness of ODD is the survival probability, based on the time of death and indication of death. NHIS-NSC provides only the death year and month; thus, we arbitrarily set up the date of death to be the last day of the month for computing the overall survival time because every claim should be earlier than the day of death.

### Statistical Analysis

Frequency and proportion were applied to the TPR and FPR *via* a confusion matrix, which is a table that is often used to describe the performance of a classifier for which the true values are known. The intervals between medical institution visits of the patients with cancer were presented as the median and interquartile range (IQR) to provide information for defining the length of a period without medical claims. The Kaplan–Meier (KM) survival curves and log-rank tests were performed to compare survival probabilities. This study design allowed a 10-year follow-up period at most. We additionally limited the follow-up periods to 3 and 5 years to show robustness by the length of the follow-up period since a 10-year follow-up period is not expected when analyzing RWD. Descriptive analysis using the box plot was performed to determine the differences between the real and operational death dates. All statistical analyses were performed using R version 4.1 (R Core Team, 2021) and the SAS Enterprise Guide (version 7.1; SAS Institute, Cary, NC, United States).

## Results

### Study Population

A total of 40,970 patients were newly diagnosed with cancer between 1 January 2006 and 31 December 2015, and 12,604 (30.76%) of them were officially recorded as dead (Table 2). In the high-mortality group, 2,896, 2,809, and 921 patients were identified as having lung, liver, and pancreatic cancers, respectively. It was confirmed that 1,917 (66.19%), 1,718 (61.16%), and 652 (70.79%) of these patients with lung, liver, and pancreatic cancers, respectively, were recorded as dead. In the middle-mortality group, 5,681, 828, and 773 patients had stomach, skin, and kidney cancers, respectively. Of the patients with stomach, skin, and kidney cancers, 1,561 (27.48%), 154 (18.60%), and 143 (18.50%), respectively, were recorded as dead. A total of 6,316 patients were diagnosed with thyroid cancer in the low-mortality group, 93 (1.47%) of whom were deceased.

**TABLE 2:** True-positive rate (TPR) and false-positive rate (FPR) according to cancer types.

Cancer type	No. of patients	No. of real death	TP	TN	FN	FP	TPR (%)	FPR (%)
All	40,970	12,604	12,185	28,006	419	360	96.68	1.27
High mortality								
Lung	2,896	1,917	1,854	970	63	9	96.71	0.92
Liver	2,809	1,718	1,671	1,083	47	8	97.26	0.73
Pancreatic	921	652	637	263	15	6	97.70	2.23
Subtotal	6,626	4,287	4,162	2,316	125	23	97.08	0.98
Middle mortality								
Stomach	5,681	1,561	1,506	4,045	55	75	96.48	1.82
Skin	828	154	141	661	13	13	91.56	1.93
Kidney	773	143	134	622	9	8	93.71	1.27
Subtotal	7,282	1,858	1,781	5,328	77	96	95.86	1.77
Low mortality								
Thyroid	6,316	93	91	6,187	2	36	97.85	0.58

TN, true negative; TP, true positive; TPR, true-positive rate; FN, false negative; FP, false positive; FPR, false-positive rate.

### Interval of Medical Institution Visits

Table 3 exhibits the median and IQR of maximum intervals between medical institution visits of patients with cancer as evidence of defining the length of a period without medical claims. We identified 39,434 patients having at least two claims during the follow-up. Among them, 11,252 deceased patients visited the medical institution again within at least 30 days (IQR 36). For alive patients, the median interval was 85 days (IQR 105). Overall, the median interval of visits for alive patients was longer in the low-mortality group, followed by the middle- and high-mortality groups. According to the cancer type, the median interval between medical institution visits of the deceased patients ranged from 22 to 45 days. In contrast, the median interval ranged from 52 to 100 days for patients alive. Additionally, of the 1,536 patients (40,970-39,434 = 1,536) who visited medical institutions once in this study, 1,352 (88.02%) patients died, not having additional claims due to death. Among the remained 184 (11.98%) alive patients, 182 (98.91%) had a follow-up period shorter than 365 days.

**TABLE 3:** The maximum intervals between medical institution visits of patients with cancer.

Cancer type	Total		Deceased patients		Alive patients	
	N^a^	Median (IQR, days)	N^a^	Median (IQR, days)	N^a^	Median (IQR, days)
All	39,434	64 (88)	11,252	30 (36)	28,182	85 (105)
High mortality						
Lung	2,631	32 (38)	1,673	27 (26)	958	52 (56)
Liver	2,583	41 (56)	1,513	30 (37)	1,070	70 (60)
Pancreatic	851	28 (36)	587	22 (19)	264	56 (83)
Middle mortality						
Stomach	5,556	72 (111)	1,453	35 (42)	4,103	91 (125)
Skin	820	63 (84)	151	45 (60)	669	66 (91)
Kidney	752	71 (94)	131	31 (33)	621	81 (121)
Low mortality						
Thyroid	6,290	99 (102)	84	36 (39.5)	6,206	100 (103)

IQR, interquartile range; N^a^, number of patients who had more than two visits, i.e., who visited medical institutions twice or more.

### Accuracy of Operational Definition of Death

**FIGURE 1:**
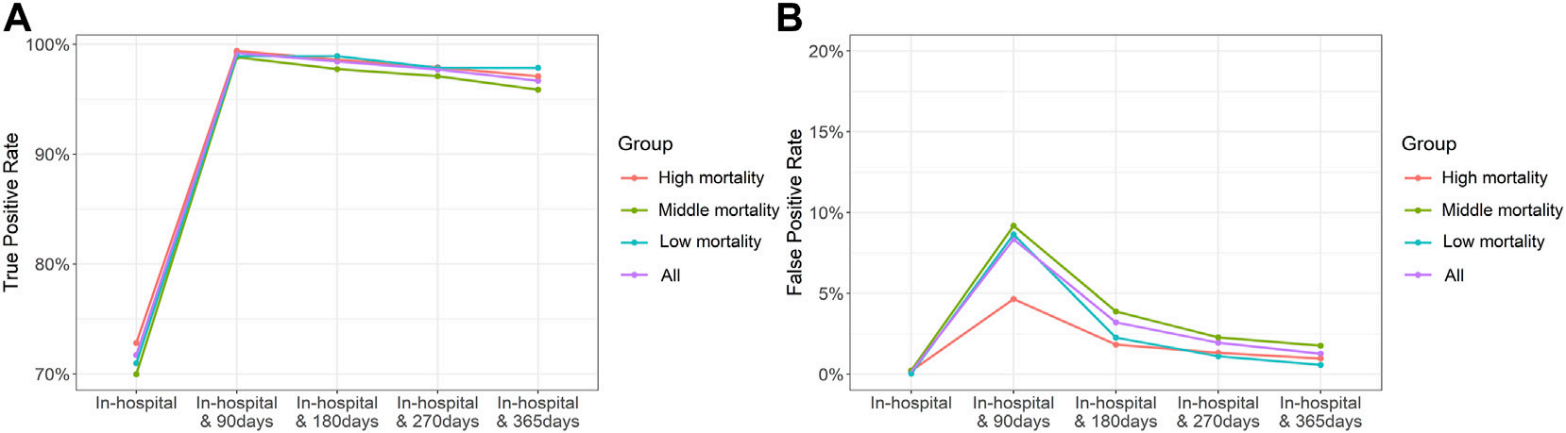
True-positive **(A)** and false-positive **(B)** rates in the duration of 0–365 days following the last claim.

The TPR and FPR according to the length of periods are presented in Figure 1 (in detail, Supplementary Tables S1, S2). In cases where only in-hospital death was considered, FPRs were close to 0 but the TPR was approximately 70%. Considering the ODD using the length of periods without medical utilization, TPRs were over 95%. TPRs were slightly decreased after 90 days from the last claim, and FPRs exhibited large variations and high values until 270 days, especially when using the length of 90 days. When using 90 days, FPRs in high-mortality cancer except pancreas were less than 5%, but those in other types of cancers were 7%. When using 365 days, TPRs were 90% over and FPRs were 3% below.

The TPR and FPR from the results determined using a combination of in-hospital deaths and cases in which there were no claims within 365 days of the last claim as an ODD are presented in Table 2. Considering all cancers, the overall TPR and FPR were 96.68 and 1.27%. The TPR indicates that 12,185 patients were identified as deceased among 12,604 patients with death records. The FPR indicates that only 360 patients were falsely identified as dead among a total of 28,366 alive patients. In the high-mortality group, the overall TPR and FPR were 97.08 and 0.98%, respectively. In the middle- and low-mortality groups, TPRs were 95.86 and 97.85% and FPRs were 1.77 and 0.58%, respectively.

When not using in-hospital death but using the length of a period without any claims, the results for 365 days revealed that TPR was 86.25% and FPR was 1.14% (Supplementary Table S2).

### Comparison of Survival Probabilities

**FIGURE 2:**
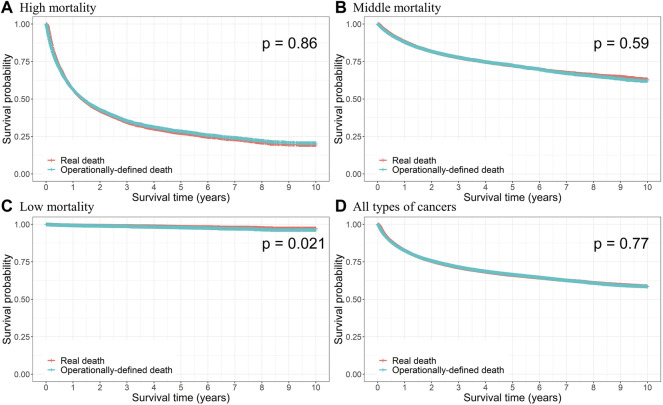
Kaplan–Meier curves of the real and operational definition of death (ODD) and log-rank test *p*-value for **(A)** high, **(B)** middle, and **(C)** low mortalities, and **(D)** all types of cancers.

For consistency in terms of the overall survival rate between real and operational deaths, we compared the KM curves for the two cases and conducted a log-rank test, and the results observed according to the mortality group are presented in Figure 2. The survival probabilities were computed for each death point, and no difference between survival curves was observed for all types of cancers, indicating that there was no significant difference between the dates of real and operational definitions of death (*p* = 0.77). No significant difference was observed for each mortality group, except for the low-mortality group (thyroid cancer; *p* = 0.021). Supplementary Figure S1 presents the KM curves and log-rank test *p*-values for specific cancer types, and the results were not different from those of the mortality groups.

When we adjusted the period for the case of no claims to 180 days, patients with lung, liver, and pancreatic cancers in the high-mortality group, and skin and kidney cancers in the middle-mortality group did not show significant differences with respect to the KM curves (Supplementary Figure S2). We considered 3- and 5-year follow-up periods as well and observed that there were no significant differences for all cancer types in both these periods (Supplementary Figures S3, S4).

Most values of the differences between the dates of real and ODD were properly distributed within the 30 days, regardless of the cancer type, which represents the accuracy of the suggested definition of death using claims data in health-related research (Supplementary Figure S5).

## Discussion

In the present study, we validated ODD *via* TPR and FPR and performed a comparison of survival curves. The TPR was found to be more than 95%, and the FPR was less than 2% in the high-, middle-, and low-mortality groups. Additionally, there was no significant difference in the survival probability between the real death and the ODD. With robustness confirmed through sensitivity analysis, we have suggested an alternative definition to indicate death and the corresponding date of death to address the absence of death-related information in claims data, especially for patients with cancer. In cases where only in-hospital death was considered, FPRs were perfectly controlled; however, TPRs were disturbed below 80%, and thus, death was not accurately identified. Therefore, researchers who use only administrative data lacking death information could also observe overall survival by defining death based on the pattern of medical utilization.

During the 10-year follow-up, the KM curves were not significantly different for all cancers, except for thyroid cancer. The incidence of thyroid cancer is increasing worldwide with the advancement of diagnostic technology (Davies and Welch, 2014; Cabanillas et al., 2016), which is well-managed enough to cause overdiagnosis, especially in South Korea (Ahn et al., 2014; Park et al., 2016). Because the mortality of thyroid cancer is very low (Statistics Korea, 2021a), it has been presumed that an operational definition could lead to an overestimation of mortality. Similarly, this study showed that cancers with relatively low mortality, including stomach, skin, and kidney cancers, had slightly lower survival probabilities in operationally defined deaths later in the follow-up period, although not significantly different. In the middle-, and low-mortality groups, FPR was more important than TPR due to the size of cases (number of death patients), and they were both controlled well (TPR middle: 95.86%; TPR low: 97.85%, FPR middle: 1.77%; FPR low: 0.58%). Conversely, cancers with poor prognosis, such as lung, liver, and pancreatic cancers, had slightly lower survival probabilities in real deaths later in the follow-up period. In this group, the TPR was relatively more important than the FPR owing to the imbalance problem, in which the proportion of dead cases was higher than that of alive cases. Our results revealed that the TPR and FPR were well-controlled. Considering 3- and 5-year follow-up periods revealed robustness, implying that this definition can be applied to other studies, regardless of follow-up periods. It can be interpreted that our ODD captures the real death well and can be used as an indication of death.

For base-case analysis, we defined death as the case when there were no claims for 365 days from the last claim. The reasoning behind adopting 365 days for ODD is presented in Figure 1. It revealed a lower FPR than the case where the claim duration was shorter. The FPR for pancreatic cancer was the highest but only slightly over 2%, and for lung, liver, and thyroid cancers was less than 1%. Additionally, the TPR was greater than 95% overall. We determined the 365-day period, which yielded the lowest FPR and acceptable TPR, as appropriate. As shown in Table 3, the medians of a maximum interval between medical institution visits were less than 100 days regardless of cancer type and the maximum of IQRs was 125 days. It implied that 90 days seemed not to be enough to define ODD. Previous studies have defined death as the case of no claims for 180 days, which is shorter than that of our study (Mealing et al., 2012; Lee et al., 2019). When the time period was reduced to 90 days, it resulted in a TPR of over 99% and also an FPR of over 8%. ODD of shorter periods significantly overestimated mortality, especially in cancers with relatively low mortality, including stomach, skin, kidney, and thyroid cancers. However, for cancers with high mortality, such as lung and liver cancers, considering a short period was also worth considering (FPR with 90 days 3.78% in lung cancer; 4.03% in liver cancer). Similarly, our definition may be more accurate for patients with advanced cancer than for patients with early-stage cancer. We also elicited TP and FP results when defining ODD using only the length of a period without any claims for 90–365 days. Although the TPR value was close to 80% for the case of no further claims within 365 days of the last claim, definitions using only the claims gap could be considered in cases of advanced cancer or cancer with low survival.

We identified why FP and FN occurred. The median interval between medical institution visits for FP patients was 252.5 days, which was approximately four times that of all patients. FP patients visited medical institutions infrequently because of which their visit interval was longer than 365 days, resulting in them being considered dead. All FN patients were confirmed to have been dead in 2015. Since the study period was only until 2015, these patients were followed up for less than 365 days of the last claim and were not operationally defined as deaths. The number of FN patients could be reduced if the researcher established a minimum follow-up period, especially a longer period than the period used for the operational definition. In this study, if the cohort entry period was maintained but the follow-up period was extended by 1 year, FN did not occur.

The overall proportion of incidence of all cancers in South Korea from 2006 to 2015 was approximately 4.06%, as per Statistics Korea (Statistics Korea, 2021a). The data used in this study provided by NHIS-NSC represented approximately 4.09%. This indicates that our data were representative datasets. The incidence rates of stomach, liver, and lung cancers were reported to be 0.59, 0.32, and 0.43% (Statistics Korea, 2021a), and those of our data were 0.56, 0.28, and 0.29%, respectively. The proportion of deaths among patients with cancer from our data was slightly lower than the reports (Statistics Korea, 2021a; Statistics Korea, 2021b) (all cancers: 30.76 vs. 35.38%; stomach cancer: 27.47 vs. 32.97%; liver cancer: 61.16 vs. 70.73%; lung cancer: 66.19 vs. 73.74%). However, this may be secondary to the sampling error. Since we utilized real death-related information of the selected patients with cancer recorded by Statistics Korea, no critical problem could affect the study to demonstrate the validity of the suggested ODD.

Despite the significance of these findings, this study had some limitations. First, we applied the ODD to patients with cancer; thus, this definition should be used carefully for other diseases in terms of generalization. However, we confirmed the TPR and FPR across various mortality groups. There is room to adapt the ODD to various diseases, especially with high mortality. Second, the selected patients might be insufficient in this study since we used diagnostic codes recorded in the claims data. However, we included patients with cancer registered under the National Health Insurance Act using critical condition codes. Also, we confirmed that the mortalities in this study were similar to the actual cancer-associated mortalities (Statistics Korea, 2021a). Third, a lack of the exact date of death and having only the information about the month of death in the database was a limitation. This was the innate limitation of the NHIS-NSC data. Thus, we provided the differences between the last day of the deceased month and the defined date in supplementary as Supplementary Figure S5. IQR of gap days was within 20 days across whole groups, even in all cancer groups not specified by cancer type. Even though we do not know the exact date, the gap days in Supplementary Figure S5 is the maximum value, which can be observed in the real world. Therefore, the difference between the real death date and the operationally-defined date would be close to zero.

Healthcare utilization can differ according to the healthcare system. However, cancer patients worldwide are mainly managed according to consensus and clinical guidelines published by the National Comprehensive Cancer Network, European Society for Medical Oncology, or American Society of Clinical Oncology. Physicians have followed up on cancer patients regularly according to guidelines even if each healthcare system to which they belong has its own system. Although survival rates differ across countries due to race, data collection, analysis, and quality, and are difficult to compare these directly (Gatta et al., 2000; Coleman et al., 2008), the rank and trend of survival rates in South Korea were similar with the United Kingdom and the United States (Cancer Research UK, 2011; Quaresma et al., 2015; Siegel et al., 2022). Therefore, the ODD we offered can be helpful when analyzing claims data to conduct outcomes research regardless of country and healthcare system.

## Conclusion

In cancer patients, defining the case of no claims within 365 days of the last claim as death can be a robust alternative for death information in claims data lacking it. By determining the appropriate ODD, this study contributes to promoting outcomes research using claim-based data that does not include death information, especially for out-of-hospital deaths.

## Data Availability

Publicly available datasets were analyzed in this study, and the data can be found here: Korean National Health Insurance Service (https://nhiss.nhis.or.kr/).
