# Supplementary material for: Optimal Indicator of Death for Using Real-World Cancer Patients' Data From the Healthcare System
Source: Front Pharmacol. 2022 Jun 16;13:906211. doi: 10.3389/fphar.2022.906211 (PMC9243505; doi:10.3389/fphar.2022.906211)
Supplement: Supplementary file 1 [file DataSheet1.docx]

Supplementary Material

Supplementary Table S1. True-positive rate (TPR) and false-positive rate (FPR) using the modified operational definition of death

| Mortalitygroup | Cancer type | In-hospital | | In-hospital  & 90days | | In-hospital  & 180days | | In-hospital  & 270days | |
| --- | --- | --- | --- | --- | --- | --- | --- | --- | --- |
|  |  | TPR(%) | FPR(%) | TPR(%) | FPR(%) | TPR(%) | FPR(%) | TPR(%) | FPR(%) |
|  | All | 71.72 | 0.16 | 99.21 | 8.35 | 98.44 | 3.22 | 97.71 | 1.96 |
| High | Lung | 71.26 | 0.31 | 99.17 | 3.78 | 98.33 | 1.33 | 97.76 | 1.12 |
|  | Liver | 73.57 | 0.18 | 99.48 | 4.03 | 98.72 | 1.56 | 97.96 | 1.10 |
|  | Pancreas | 75.46 | 0.00 | 99.85 | 10.41 | 99.08 | 4.83 | 98.16 | 2.97 |
| Middle | Stomach | 70.60 | 0.29 | 98.98 | 9.81 | 98.14 | 4.17 | 97.57 | 2.40 |
|  | Skin cancer | 61.04 | 0.15 | 98.05 | 7.12 | 94.16 | 3.12 | 93.51 | 2.37 |
|  | Kidney | 72.73 | 0.00 | 97.90 | 7.30 | 97.20 | 2.86 | 95.80 | 1.43 |
| Low | Thyroid | 70.97 | 0.05 | 98.92 | 8.65 | 98.92 | 2.27 | 97.85 | 1.12 |

FPR, false-positive rate; TPR, true-positive rate;

Supplementary Table S2. True-positive rate (TPR) and false-positive rate (FPR) using the modified operational definition of death without in-hospital death

| Mortalitygroup | Cancer type | 90 days | | 180 days | | 270 days | | 365 days | |
| --- | --- | --- | --- | --- | --- | --- | --- | --- | --- |
|  |  | TPR(%) | FPR(%) | TPR(%) | FPR(%) | TPR(%) | FPR(%) | TPR(%) | FPR(%) |
|  | All | 96.42 | 8.21 | 93.18 | 3.08 | 89.95 | 1.83 | 86.25 | 1.14 |
| High | Lung | 96.14 | 3.58 | 92.59 | 1.12 | 88.99 | 0.92 | 85.03 | 0.72 |
|  | Liver | 97.15 | 3.85 | 94.18 | 1.37 | 90.86 | 0.92 | 87.14 | 0.55 |
|  | Pancreas | 96.47 | 10.41 | 92.64 | 4.83 | 89.11 | 2.97 | 85.74 | 2.23 |
| Middle | Stomach | 96.86 | 9.56 | 94.30 | 3.93 | 91.29 | 2.16 | 88.02 | 1.58 |
|  | Skin cancer | 93.51 | 6.97 | 88.31 | 2.97 | 86.36 | 2.23 | 80.52 | 1.78 |
|  | Kidney | 94.41 | 7.30 | 91.61 | 2.86 | 88.81 | 1.43 | 83.92 | 1.27 |
| Low | Thyroid | 96.77 | 8.60 | 93.55 | 2.22 | 87.10 | 1.08 | 84.95 | 0.53 |

FPR, false-positive rate; TPR, true-positive rate


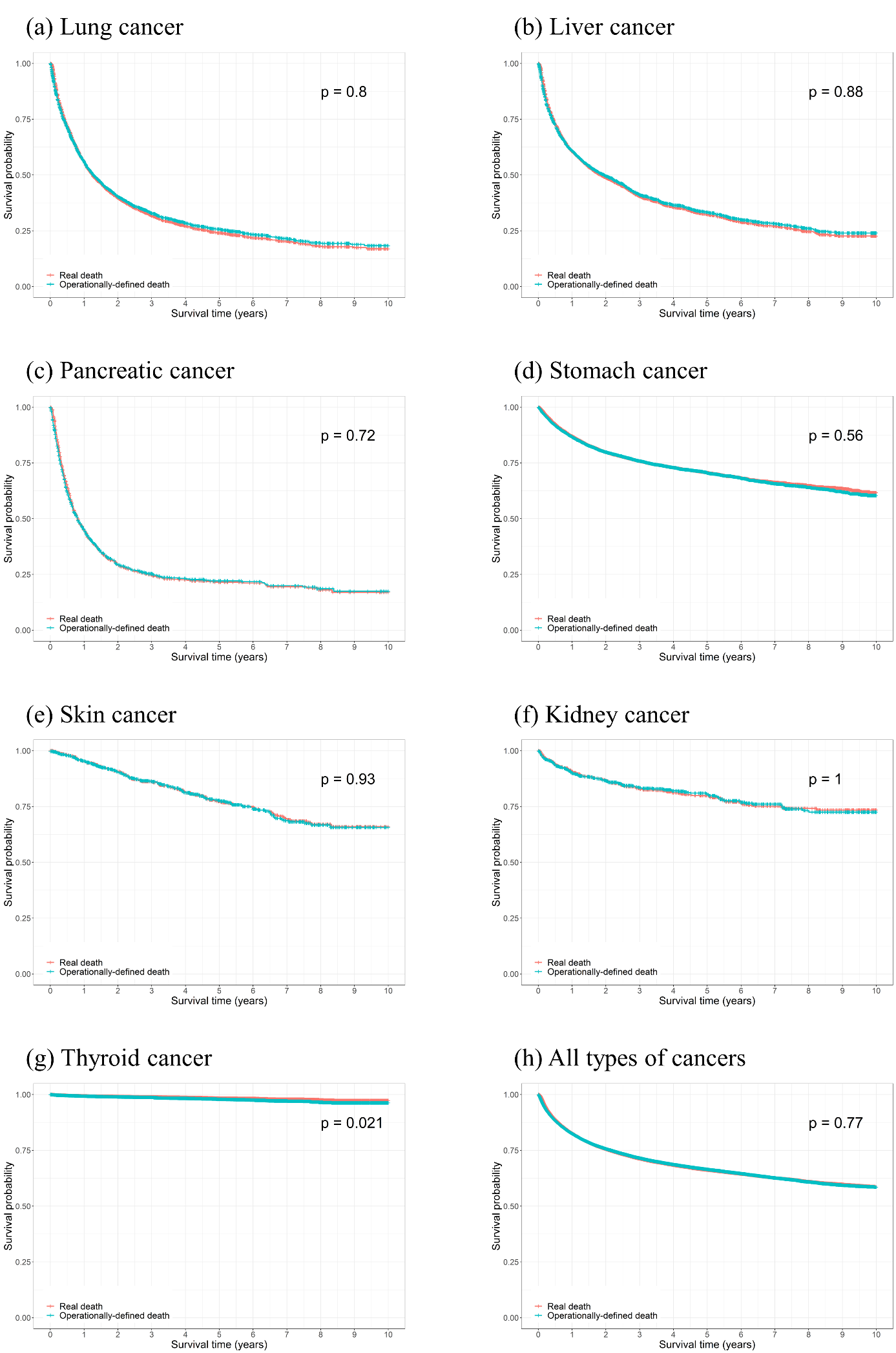


Supplementary Figure S1. Kaplan-Meire curves of the real and operational definition of death; (a) lung cancer, (b) liver cancer, (c) pancreatic cancer, (d) stomach cancer, (e) skin cancer, (f) kidney cancer, (g) thyroid cancer, and (h) all types of cancers. p=the *P*-value for the log-rank test


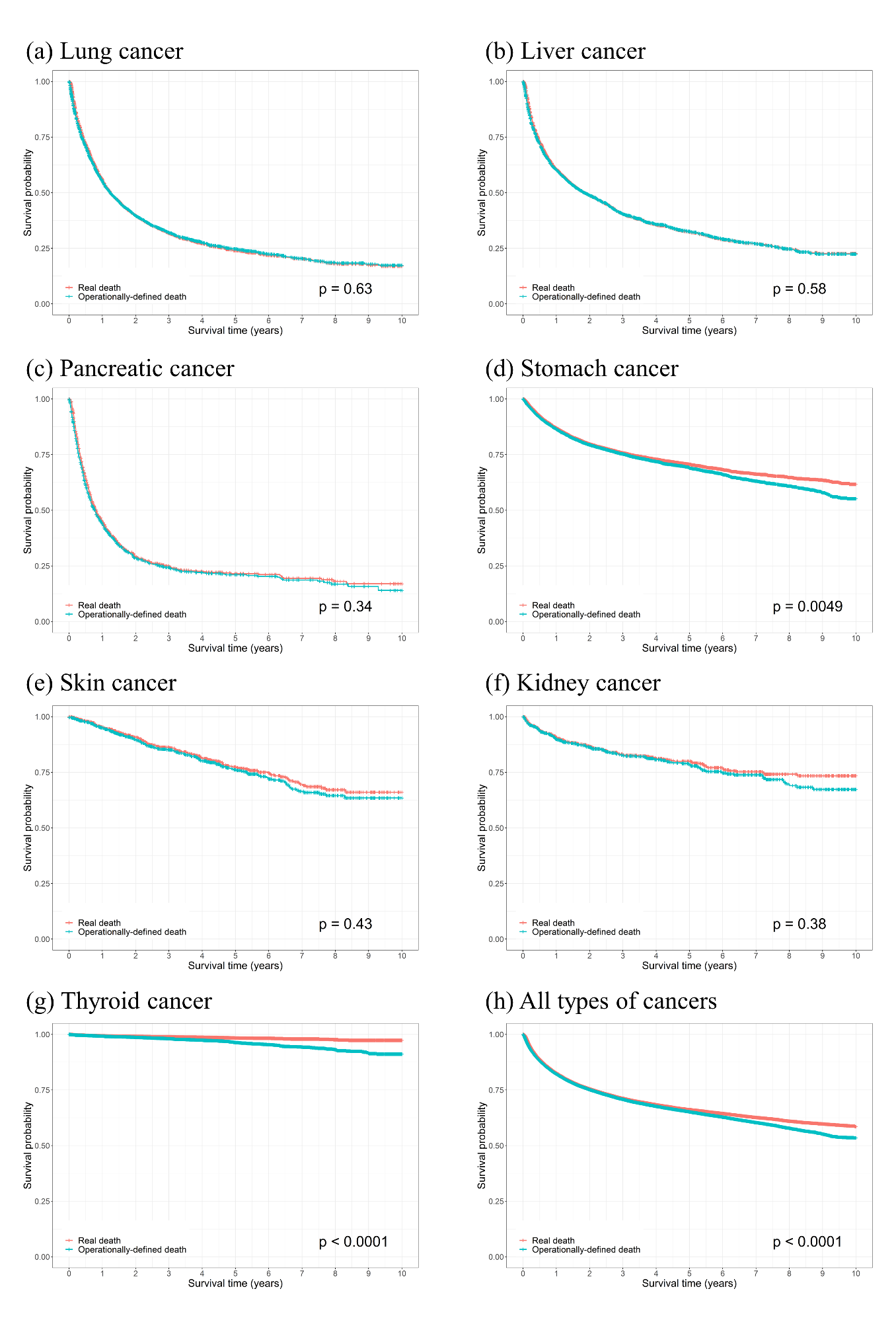


Supplementary Figure S2. Kaplan-Meire curves of the real and modified operational definition of death using a gap of 180 days; (a) lung cancer, (b) liver cancer, (c) pancreatic cancer, (d) stomach cancer, (e) skin cancer, (f) kidney cancer, (g) thyroid cancer, and (h) all types of cancers. p=the *P*-value for the log-rank test


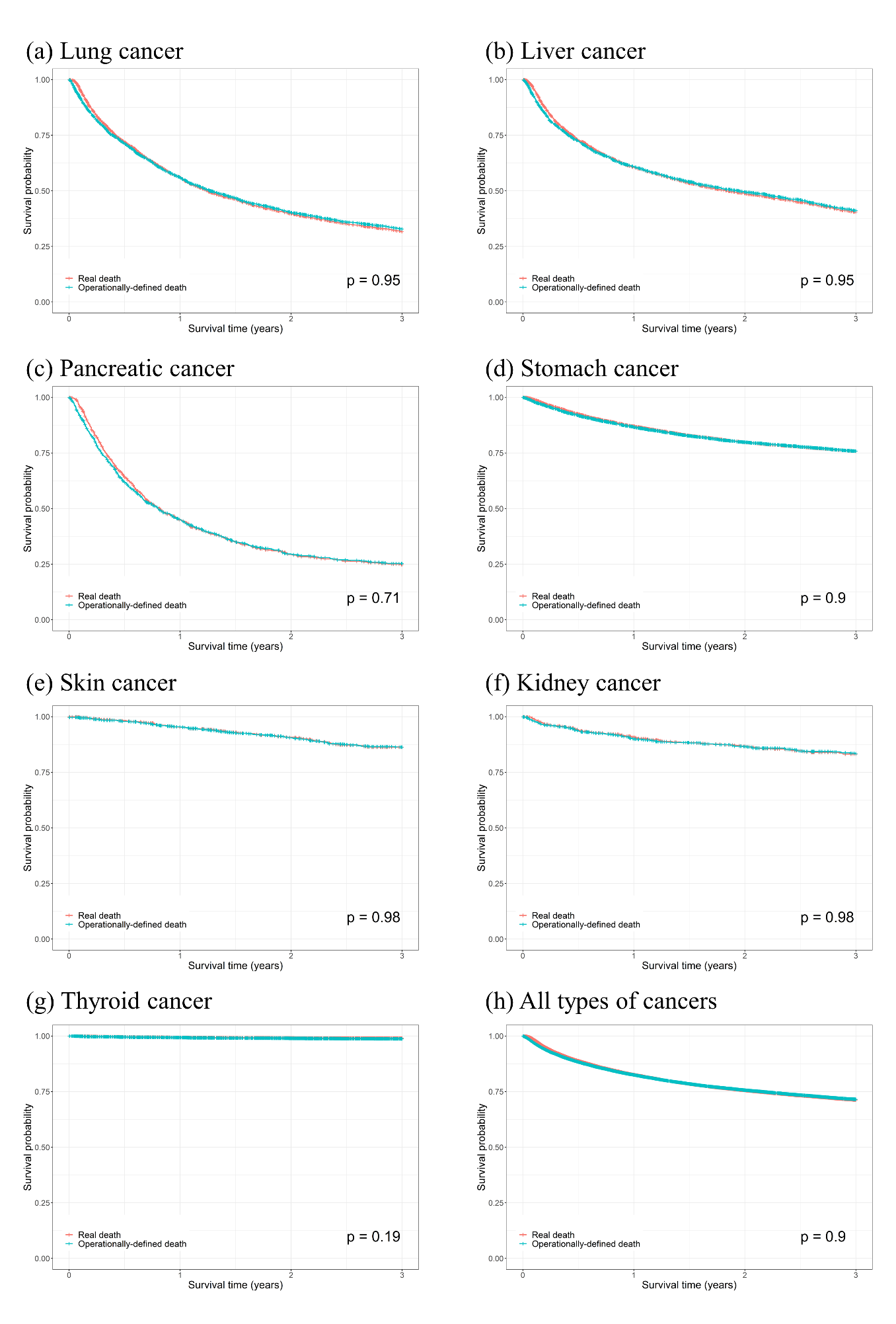


Supplementary Figure S3. Kaplan-Meire curves of the real and operational definition of death (3-year of the follow-up period); (a) lung cancer, (b) liver cancer, (c) pancreatic cancer, (d) stomach cancer, (e) skin cancer, (f) kidney cancer, (g) thyroid cancer, and (h) all types of cancers. p=the *P*-value for the log-rank test

**
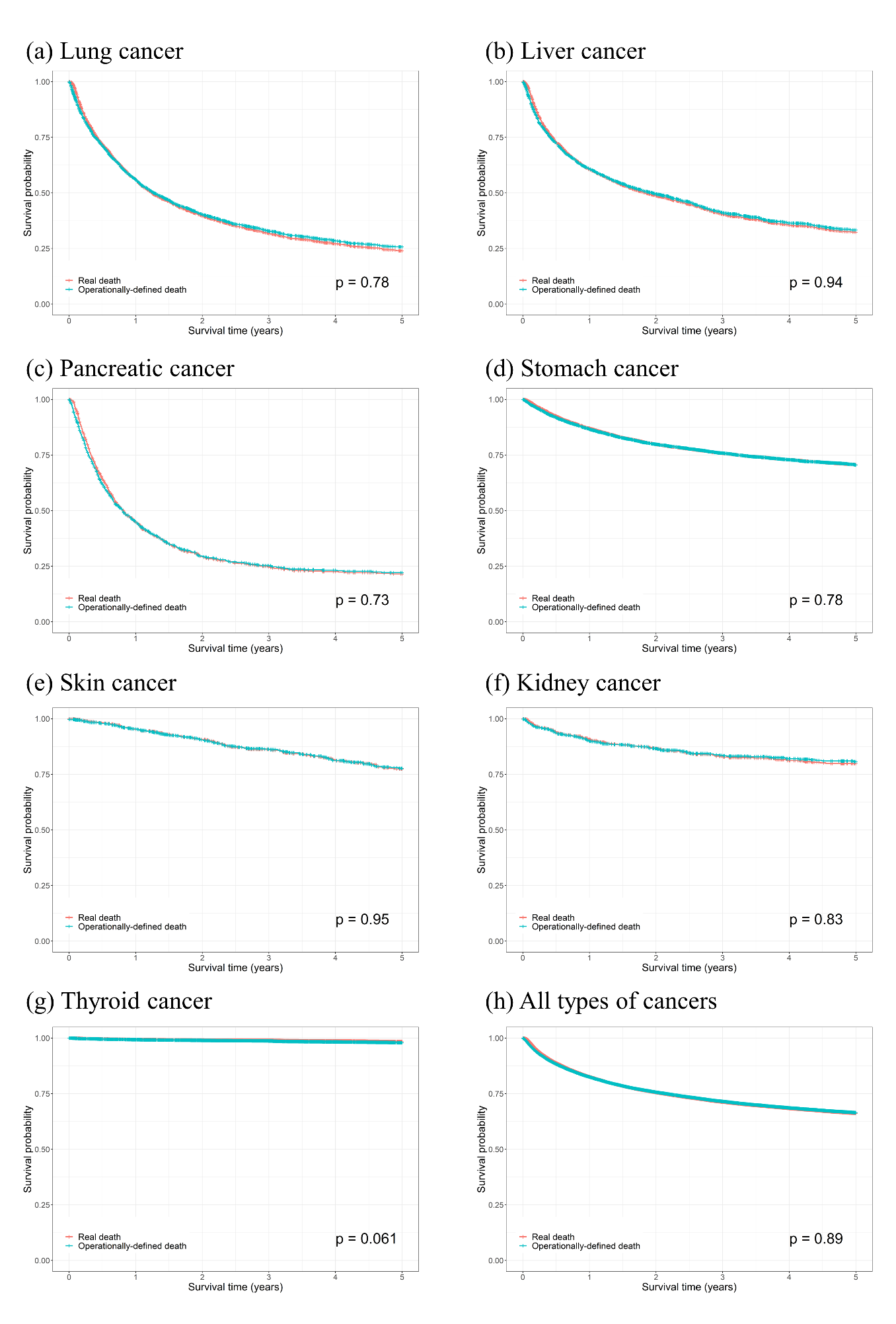
**

Supplementary Figure S4. Kaplan-Meire curves of the real and operational definition of death (5-year of the follow-up period); (a) lung cancer, (b) liver cancer, (c) pancreatic cancer, (d) stomach cancer, (e) skin cancer, (f) kidney cancer, (g) thyroid cancer, and (h) all types of cancers. p=the *P*-value for the log-rank test


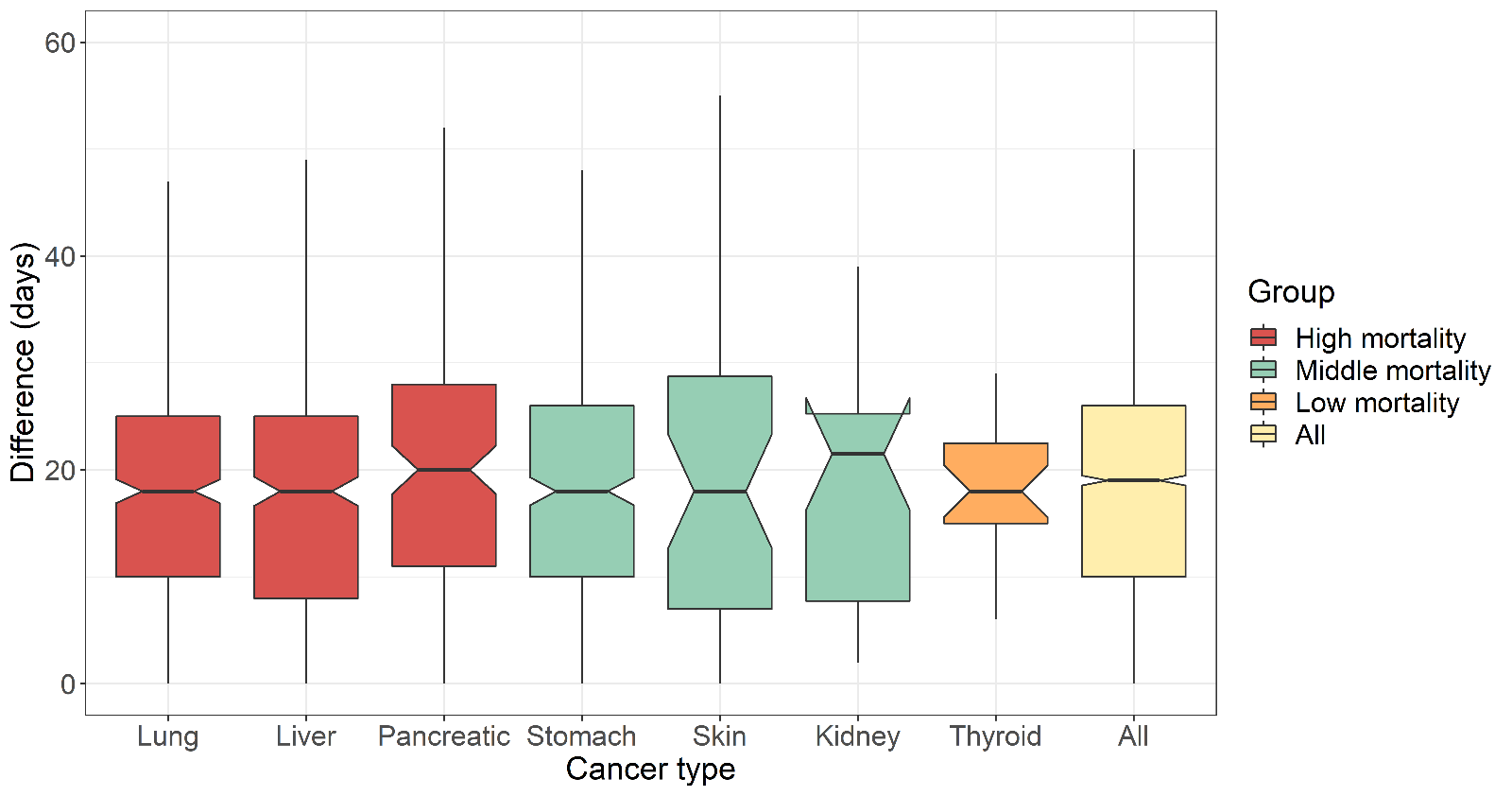


Supplementary Figure S5. Box plot for the difference between dates of the real and operational deaths according to cancer types, when we assumed real dates of death on the last day of each month
